# Autonomous ‘self-driving’ laboratories: a review of technology and policy implications

**DOI:** 10.1098/rsos.250646

**Published:** 2025-07-16

**Authors:** Alexander V. Tobias, Adam Wahab

**Affiliations:** ^1^ Department of Biotechnology and Life Sciences, The MITRE Corporation, McLean, VA, USA

**Keywords:** autonomous science, self-driving laboratories, artificial intelligence, closed-loop experimentation, cloud laboratories, intellectual property, autonomous chemistry, autonomous materials science, autonomous biology

## Abstract

This article reviews and provides perspective on the emerging technology of autonomous, ‘self-driving’ laboratories (SDLs) that combine artificial intelligence (AI) and laboratory automation to perform research in chemistry, materials science and biological sciences. Today’s most capable SDLs automate nearly the entire scientific method, from hypothesis generation, experimental design, experiment execution and data analysis, to drawing conclusions and updating hypotheses for subsequent rounds of optimization or discovery. ‘Cloud labs’ offer subscription-based remote-control access to experimental capabilities. Reports of AI-directed experiments executed in cloud labs are appearing in the literature, previewing a democratization of science that intrigues but inspires concern. Indeed, SDLs have potential implications for society far beyond the academy. Inventions emerging from AI-driven science pose a grand challenge, as patent laws across the world recognize only human inventors. If the inventions they generate remain unpatentable, funding for SDLs may be constrained. SDLs raise safety and security concerns. We deem them surmountable with a proactive approach, ultimate human accountability and robust cybersecurity measures. Finally, we estimate the impacts of SDLs on the technical labour force. Our analysis suggests that SDLs may displace some scientific roles but are likely to create many new opportunities.

## 1. Introduction

The advancement of human civilizations has been driven by the development of ever more powerful and useful tools. Seminal inventions from the abacus to the personal computer have enabled step-change leaps in the speed, power and accuracy with which societies perform the very work of scientific discovery and technology development. Foundational tools tend to engender positive feedback loops that convert once arduous or laborious undertakings into routine, automated and often largely hidden tasks.

The release of the large language model (LLM) ChatGPT in 2022 launched an unprecedented wave of artificial intelligence (AI) [1] directly into the hands of the public. This wave has already impacted nearly all facets of society, including science and technology development. Author and founder of the AI company DeepMind, Mustafa Suleyman, remarked that the hallmark distinction between artificial intelligence and all previous technologies is that AI systems can self-teach, improve themselves and perform many complex tasks and workflows *autonomously* [2].

The scientific method can be viewed as a cycle of steps. Researchers conceive of questions and formulate testable hypotheses based thereon. Experiments are designed to test the hypotheses. The experiments are conducted, and the ensuing data are analysed and processed into results that, ideally, point toward acceptance or rejection of the hypothesis. As the results are disseminated throughout the research community, they may inspire follow-on ideas, questions and hypotheses. Thus, additional turns of the cycle follow and science advances. Technology has accelerated, routinized, reduced costs and otherwise transformed key steps of this cycle. Computers and software have dramatically improved and democratized the analysis and processing of data and the ability to run simulations to better understand or even replace several types of experiments. In some disciplines such as biotechnology, robotics and precision machines have substantially increased the number of experiments that can be performed per unit of space or time and reduced human labour to a small fraction of that required for manual workflows; however, the conception of research questions and hypotheses have, until the last few years, been the exclusive domain of highly educated humans. These tasks were simply too complex, subtle, or required too much knowledge or understanding to even be considered tractable by a machine.

A veritable movement of autonomous science is underway that is beginning to influence change in these concepts. Researchers in the chemical, materials and biological sciences are combining laboratory automation with AI to create new systems capable of performing all the physical and intellectual steps of the scientific method. In the literature, these systems are variously called ‘robot scientists’, ‘AI scientists’, or, by analogy to self-driving vehicles, ‘self-driving labs’ [3]. Despite the numerous and profound dissimilarities between performing science and controlling a vehicle, the latter name, abbreviated ‘SDL’ (plural: ‘SDLs’), appears to be the most common at present. For example, the Acceleration Consortium, a leading global network devoted to autonomous science, uses the term ‘self-driving labs’ and ‘SDLs’ throughout its webpages [4].

Self-driving labs have emerged from obscure and clunky academic curiosities into demonstrably useful tools for contemporary science. SDLs are already leading to the discovery of molecules and materials with commercial potential. Section 2 describes different types of SDLs reported in the technical literature and the media and highlights some of the most impactful research performed with (or by) the technology. Beyond serving as a tool for assisting or accelerating research, AI systems and SDLs can and have independently generated novel inventions [5]. This has led to one of the most contentious questions around the technology: how can and how should legal systems handle the intellectual property (IP) generated by AI and SDLs? Section 3 reviews this issue and provides some suggestions for how IP law could be updated for the age of AI inventorship.

Technologies as powerful, general-purpose and potentially transformative as AI engender fears and concerns among certain experts and members of the public [2]. When technical disciplines such as robotics, chemistry and biology are combined with AI, as they are with SDLs, frightening scenarios are easily conjured based on prior incidents, science fiction and our imaginations. The safety and security issues surrounding SDLs are the subject of §4. Will SDLs replace scientists the way AI may disrupt professions across the economy? §5 investigates this question.

This report is not intended to serve as a comprehensive review of the SDL field. For that, we recommend the review by Tom *et al.* [6]. Rather, we focus herein on influential developments and contemporary issues within and adjacent to the field to broaden awareness and provoke thought and discussion about its past, present and potential future.

## 2. Types, examples and significance of SDLs

### 2.1. Levels of autonomy

Researchers have proposed a classification system, adapted from the automation levels for self-driving vehicles created by the Society of Automotive Engineers [7], to evaluate the degrees of autonomy of scientific automation systems and assistive technologies [8]. This classification system for scientific research autonomy is described below and summarized in table 1.

**Table 1.** Levels of autonomy for SDLs [8–10].

autonomy level	name	description	examples
1	assisted operation	machine assistance with laboratory tasks	robotic liquid handlers, data analysis software
2	partial autonomy	proactive scientific assistance, e.g. protocol generation	Aquarium [11]
3	conditional autonomy	minimum to qualify as an SDL. Autonomous performance of at least one cycle of the scientific method. Interpretation of routine analyses, testing of supplied hypotheses. Require human intervention only for anomalies	iBioFab [12], Mobile Robot Chemist [13]
4	high autonomy	an hypothesis tester capable of automating protocol generation, experiment execution, data analysis and results-driven hypothesis adjustment	Adam [14], Eve [15], MicroCycle [16] 01/11/2024 20:32:00
5	full autonomy (AI researcher)	full automation of the scientific method	not yet achieved

Level 1 is marked by machine assistance with defined tasks. For example, liquid handling robots may dispense and manipulate reagents for experiments, or computers may facilitate calculations and data analysis. In Level 2, at least one ‘intellectual’ aspect of the scientific method has been automated. Use of predictive machine learning (ML) or a dynamic workflow planning tool such as Aquarium [11] falls within Level 2.

Level 3 represents an inflection in autonomy and the classification of most present-day SDLs. Level-3 scientific systems can autonomously perform multiple cycles of the scientific method. These systems interpret and learn from the results of a previous cycle to inform the designs of the next. Level-3 systems are considered ‘conditionally autonomous’ in that they require human intervention only for anomalous cases.

Level-4 systems are capable of highly autonomous research. They are comparable with skilled lab assistants and can automate protocol generation, execution, data analysis and drawing of conclusions. At this level, after a human scientist provides initial hypotheses, goals and plans, the SDL can modify and update the hypotheses as it proceeds through cycles of the scientific method. To date, Level 4 is the maximal autonomy reached by SDLs described in the literature. Adam [14] and Eve [15] are two examples. Adam could design and execute experiments to evaluate gene-function hypotheses in yeast (see §2.4). Eve designed and performed experiments to identify hit compounds to treat malaria. Additional examples of Level-4 SDLs in other fields are presented below.

A Level-5 SDL functions as a full-fledged (artificially) intelligent research scientist. The human manager need only set high-level research goals and the SDL would autonomously design and perform multiple cycles of the scientific method to achieve them. The SDL is ‘in charge’ and the humans merely serve its needs (for things like maintenance and consumable replenishment) and ultimately receive its results [8]. This level of SDL has not yet been realized.

An alternative SDL classification system has been proposed that separately considers ‘hardware autonomy’ (physical automation) and ‘software autonomy’ in determining an overall SDL autonomy level [6]. Table 2 summarizes this two-dimensional framework. The hardware autonomy dimension is straightforward. The four levels of autonomy correspond to the extent to which experiment execution is automated: no automation (Level 0), isolated single tasks or experiments (Level 1), multiple successive tasks or experiments constituting a workflow (Level 2), or fully automated experimentation with only manual restocking, resetting and maintenance (Level 3).

**Table 2.** SDL hardware and software autonomy levels [6]. Abbreviations: SS, search space; ES, experiment selection.

	hardware autonomy level			
software autonomy level	manual experiment 0	automated single task or experiment 1	automated workflow 2	automated laboratory 3
human ideation *SS: human* *ES: human* 0	level 0	level 1	level 2	
single cycle *SS: human* *ES: computer* 1	level 1	level 2	level 3	
multiple ‘closed-loop’ cycles *SS: human* *ES: computer* 2	level 2	level 3	level 4	
generative *SS: computer* *ES: computer* 3				level 5

In the software autonomy dimension, the levels are gauged by capability for multiple ‘closed-loop’ cycles of autonomous experimentation and whether decisions about ‘search space’ and ‘experiment selection’ are made by humans or computers. These concepts are most easily explained in the context of optimization experiments. ‘Search space’ refers to the global set of variables and their values determined to be ‘within bounds’ for an experiment, whereas ‘experiment selection’ corresponds to the experimental runs (combinations of variable values or settings) chosen for execution in a cycle of the optimization effort.

The overall SDL autonomy level is then determined by the rubric shown in table 2. Of note is that Level-4 SDLs must be at least Level-2 in both software and hardware, and a Level-5 SDL, which has not yet been demonstrated, must be Level-3 in both dimensions [6].

With five levels of overall autonomy influenced by software and hardware considerations, the one-and two-dimensional autonomy scales are comparable. We appreciate the two-dimensional SDL autonomy framework for its explicit differentiation of software and hardware autonomy, which represent qualitatively different scientific and engineering challenges and contributions. Software autonomy is concerned with the intellectual aspects of experiments: designs, decisions and analyses. Hardware autonomy, on the other hand, is focused on highly capable and independent laboratory robotics and automation, for example, fully unattended operation, execution of complex and lengthy experimental protocols, or self-directed navigation through a laboratory. However, while laboratory robots may perform tasks more quickly, efficiently, repeatably, continuously, or in a smaller, larger, or otherwise different form factor, they almost never perform tasks that would be outright impossible for a human laboratory worker. Consequently, for the application of SDLs to the advancement of science, software autonomy is preeminent, because progress in chemistry, materials or biology is most impacted by the intellectual content of experiments. We revisit this idea in §6.

### 2.2. Chemistry

The groundwork of SDL technology was laid decades ago with early advancements in AI. The DENDRAL project at Stanford University in the 1960s developed the first example of ML software capable of scientific hypothesis formation [17]. DENDRAL was programmed with a set of chemistry rules that enabled it to predict chemical structures from input mass spectrometry data. Meta-DENDRAL, developed in the 1970s, augmented DENDRAL with a form of closed-loop learning [18]. Meta-DENDRAL was provided with input molecular structures and their corresponding mass spectrometry data. The model identified fragmentation patterns and developed heuristics about bond breaking, which led to improvements in mass spectrometry-based determination of molecular structures [18]. As additional data were input into Meta-DENDRAL, the software proved capable of learning and honing its structure-prediction abilities. These ML advancements, coupled with advancements in automation, paved the path for subsequent development of many chemistry SDLs. Pioneering examples in this sub-domain are described herein.

Although there are reports of rudimentary SDLs developed by pharmaceutical companies in the 1970s, the first published example of a chemistry SDL for reaction optimization dates to 1988 [19]. In this pioneering endeavour, the researchers developed a platform featuring a robotic arm to transport and manipulate materials and an ultraviolet-visible absorbance spectrophotometer to monitor the progress of reactions. This chemistry SDL autonomously optimized the reactions between phosphotungstic acid and various drug molecules. The system could measure product yields and increase them by adjusting the quantity of phosphotungstic acid or reaction time. It is remarkable that this SDL, which meets the criteria for Level-3 autonomy, was developed decades ago. This concept of analysis-based chemical reaction optimization has been applied to numerous other lab instruments and techniques.

In 1982, the first Level-3 SDL for post-reaction chemical separation was reported [20]. This SDL utilized high performance liquid chromatography (HPLC) to monitor and fractionate mixtures of organic compounds. The SDL would analyse the results and autonomously adjust the mixture of mobile phase solvents to optimize separation of the compounds.

SDL research efforts declined precipitously through the 1990s, a period that came to be known as an ‘AI winter’ [21] that experienced reduced interest and investment resulting from disappointment and failure to deliver on lofty promises.

A recent chemistry SDL with notable hardware complexity was also developed by University of Liverpool researchers [22]. This SDL performs solid-state synthesis, which involves high-temperature reactions of solid powders instead of mixing liquid reagents under more moderate conditions. A laborious workflow was autonomously performed by three multipurpose robots, which included crystal growth, preparation of crystal samples and powder X-ray diffractometry analysis. The activities of the three robots are orchestrated by ARChemist, a bespoke ‘system architecture’ software. As this study was a proof-of-principle experiment, only one experimental cycle was conducted to demonstrate the concept. The authors are augmenting the machine learning (ML) algorithms of the SDL to improve prediction of the crystal polymorph(s) (alternative three-dimensional arrangements of the same molecule) formed under specific crystallization conditions. The authors deliberately designed this system to be modular and readily adapted to conduct a variety of other solid-state chemistry workflows.

Researchers at the Lawrence Berkeley National Laboratory recently reported a chemical SDL for autonomous solid-state synthesis of inorganic powders [23]. This Level-4 SDL, named A-Lab, combines literature data, ML algorithms and active learning to autonomously plan and synthesize input target compounds, perform X-ray diffraction analysis, and interpret the results of the experiments. A-Lab initially proposes up to five synthesis routes for each target product. The system then applies an active learning route optimization algorithm to identify potentially improved reaction pathways. The hardware consists of three integrated stations for precursor preparation, heating, and product handling and characterization. A-Lab was able to successfully synthesize 71−74% of the target materials it was presented. The scientists attribute this high success rate to the software’s extensive ‘knowledge’ of chemical properties and synthesis heuristics from the literature and databases such as the Materials Project [24], plus its ability to actively learn from its own results. Providing A-Lab with an extensive knowledge base and equipping it with learning abilities mirrors the way human scientists are taught content and thinking skills that eventually enable them to perform original research.

Researchers at IBM have developed an autonomous chemical synthesis SDL, RoboRXN [25], that integrates cloud computing, AI and commercial automation. The platform is powered by multiple ML models that enable automated conversion of chemical preprint literature into structured knowledge graphs and complete automation of a chemical synthesis plan. The researchers demonstrated RoboRXN by using it to discover sulfonium photoacid generator compounds with desirable properties. Upon discovery of ideal candidates by the models, RoboRXN generated synthetic routes to them via retrosynthetic analysis. The final down-selected candidate, a substituted variant of a dialkylphenylsulfonium core, was then autonomously synthesized by the integrated system. RoboRXN is an example of a Level-3 software autonomy SDL that can independently explore a chemical search space, propose new hypotheses, design experiments, execute them and evaluate the results to accept or reject the hypotheses.

A chemistry SDL with a similar level of autonomy and complexity was developed by researchers in the Jensen lab at the Massachusetts Institute of Technology [26]. This SDL is a closed-loop autonomous molecular discovery platform that designs new molecules with key target properties, synthesizes them, measures the properties and leverages the resulting data as it reruns the cycle, leading to improved versions of the molecules. The SDL features a custom Master Control Network (MCN) orchestrator module that controls a liquid handler with heater-shaker, an HPLC with automated fraction collection, a robotic arm, plate reader, storage unit and high-temperature reactor. The SDL also includes two databases: one for experimental designs and one for experimental results. The properties subject to optimization by the system are wavelength of maximum absorption, lipophilicity (partition coefficient) and rate of photo-oxidative degradation.

Table 3 summarizes several other notable chemistry SDL publications. As databases and learning algorithms continue to develop, the accuracy and sophistication of SDLs like those described in this section will no doubt further improve.

**Table 3.** Additional chemistry SDL publications of significance.

description	significance to SDLs	chemistry	hardware	software	reference
early example (2007) of a modern closed-loop SDL with autonomous reaction synthesis optimization	autonomous adjustment of reactant flow rates and the reaction temperature to optimize nanoparticle synthesis conditions	cadmium selenide nanoparticles were generated by mixing cadmium oxide and selenium solutions	chip-based continuous flow microreactor with online fluorescence detection	control algorithm reduced each spectrum to a scalar ‘dissatisfaction coefficient’ to be minimized. Noise-tolerant global search algorithm autonomously selected injection rates and temperature to yielded optimum predicted fluorescence intensity	[27]
synthetic chemistry SDL (2022) designed to optimize reaction conditions	sampled large parameter space of 11 substrate pair combinations, 7 catalysts, 3 solvents, 2 bases and 2 reaction temperatures	obtained Suzuki–Miyaura coupling reaction yield substantially better than previous widely used condition	incubated and stirred multi-vial reactor system. Automated Schlenk system to purge oxygen. Manual intervention required to dispense liquid reagents and load vials into machine. Post-reaction analysis was manual	ML model of reaction yield trained with results of initial designed experiment	[28]
synthetic chemistry SDL (2018) inspired by human chemical intuition	rapid, autonomous exploration of a substrate-pair reactivity variable space	discovered 4 novel Suzuki–Miyaura coupling reactions	bespoke chemical-handling robot, in-line nuclear magnetic resonance and infrared spectroscopy	ML algorithms trained to predict reactivity of reagent combinations	[29]
a mobile chemistry SDL designed to automate the researcher instead of the instruments (2020)	autonomously performed 688 experiments in 8 days. Can be adapted to function in other conventional laboratories	discovered improved photocatalysts for production of H_2_ gas from water	a dexterous robot that moves throughout the lab and operates equipment. Performs solid and liquid dispensing, vial capping and uncapping	performs empirical batched Bayesian search and optimization without a model of chemical theory	[13]
*Synbot* (2023) autonomously plans executes, and iteratively refines chemical synthesis schemes	especially large and well-equipped SDL for optimization of synthetic reaction schemes. Advanced software architecture	autonomously designed, executed and optimized the synthesis of several compounds by, e.g. substituting solvents and catalysts	large (9.35 × 6.65 m) elaborate assemblage of interconnected instruments for material storage and handling, sample preparation, chemical reaction agitation and incubation, and analytical characterization	three software ‘layers’: AI layer to compose synthesis routes, analyse data and make decisions. Robot software layer generates automation scripts. Robotic layer executes experiment and collects data	[30]

### 2.3. Materials science

A widely accepted distinction between chemicals and materials is that chemical compounds become materials when they demonstrate some utility [31]. Approximately 20% of the industrial base and 70% of technical innovations rely on advanced materials [32]. Many countries have resolved that investing in advanced materials development is of strategic importance and have established multi-agency initiatives such as the United States’ Materials Genome Initiative [33] and multinational syndicates such as the European Advanced Materials 2030 Initiative [34]. Key hallmarks of these advanced materials initiatives are the development of materials acceleration platforms (MAPs), which function as Level-3 or higher SDLs for advanced materials discovery [35]. MAPs autonomously design, synthesize, characterize, and test novel candidate materials in repeated, closed-loop cycles. A few notable or pioneering MAPs are described herein and in table 4.

**Table 4.** Additional materials science SDL publications of significance.

description	significance to SDLs	materials science	hardware	software	reference
autonomous research system for additive manufacturing (three-dimensional printing, 2021)	first three-dimensional printer-based MAP	system autonomously modulated four printing parameters to match a target specification	syringe extruder with machine vision. Autonomously adjustable extruder parameters: prime delay, print speed, x-position, y-position	in-line automated image capture and analysis with direct feedback to a ML planner to optimize three-dimensional printing parameters	[36]
semi-autonomous MAP for adhesive materials (2022)	multi-step workflow: formula preparation (required human intervention), substrate cleaning, test specimen creation, specimen curing, adhesive strength testing, data analysis and ML-based formula modification	semi-autonomously optimized base-to-accelerant ratio of epoxy formulations to maximize bond strength	four-axis robotic arm that moves aluminium dollies through various stations. Camera to assess cleaning step. Developed special automated pull test method	custom graphical user interface coded in Python. Bayesian optimization algorithm designed the next set of formulations to test	[37]
MAP for perovskite crystal discovery (2020)	platform began as a standard robotic workcell, was converted into an SDL with the addition of ML features, then further amended with remote access features	discovered novel chiral perovskite crystals by adjusting reaction temperature and perovskite nanocrystal solution concentration	robot arm, syringe pumps, microfluidic reactor with *in situ* spectrometer and temperature controller, circular dichroism spectrometer	custom automation management system coded in Python. Optimization by reinforcement learning. Sophisticated security layer	[38]

Researchers in the Berlinguette laboratory at the University of British Columbia have developed a modular SDL named Ada that functions as a thin-film MAP [39]. Ada autonomously optimizes the optical and electronic properties of thin-film materials. The team demonstrated the capabilities of Ada by enhancing the hole mobility of an organic material used in perovskite solar cells. The autonomous workflow involves synthesizing the thin-film material, measuring several of its physical properties, calculation of hole-mobility parameters based on the data and running a Bayesian optimization algorithm to decide the inputs of the next experiment. Ada was the first MAP to autonomously optimize composition and processing parameters for thin films. The platform’s modularity was demonstrated in a subsequent project in which Ada was upgraded with the addition of a six-axis robotic arm and enhanced ML algorithms for optimizing multiple objectives [40]. The improved Ada was used to optimize the processing temperature and resulting conductivity of palladium thin films. The result was discovery of new synthesis conditions more than 50°C below the prior state of the art.

The following three examples highlight interconnected SDLs spanning multiple laboratories, facilities and geographic locations. The global scientific community has always been highly networked and an early adopter of electronic communication technologies. The ‘uber-SDLs’ presented forthwith represent exciting variations on traditional inter-laboratory collaboration, featuring information standardization, experimental specialization and automation, and clever combinations of artificial intelligence and human ingenuity.

A research team at the University of Erlangen-Nuremburg built a materials science SDL called AMANDA (Autonomous Materials and Device Application Platform) [41]. AMANDA is a platform for distributed materials research composed of a central software hub and several MAP ‘spokes’. The AMANDA team demonstrated its capabilities with the LineOne MAP, an SDL designed to produce solution-processed thin film devices. Closed-loop screening with AMANDA LineOne spawned organic photovoltaic cells with a high level of power conversion efficiency. The steps in this material development cycle were chemical synthesis, precursor creation, component addition, functional quantification and stress testing. The LineOne MAP is composed of 150 automated instruments spanning 37 different device types. The architecture and user interface of AMANDA permit users to create virtually connected labs with cross-platform data integration.

A large team of collaborating researchers from at least nine institutions across three continents recently established a distributed SDL and used it for closed-loop discovery of organic laser emitters [42]. This team created a central network to coordinate and apportion the project workflow across five SDLs. A central AI module designed, planned and scheduled a ‘multi-thread’ experimental scheme for the geographically distributed synthesis and optical characterization of organic solid-state laser compounds. This coordinated, asynchronous effort reduced workflow bottlenecks and allocated tasks to appropriately specialized facilities. Ultimately, this herculean effort was successful at discovering 21 new organic solid-state materials with state-of-the-art laser performance properties.

Research groups in five countries developed an innovative distributed MAP for battery electrolyte development [43]. This SDL has a truly decentralized architecture featuring a ‘brokering’ software system called FINALES (Fast Intention-Agnostic Learning Server) that coordinates the overall workflow among the geographically separated MAPs. With this design, no individual MAP performs all workflow steps, but each contributes its capabilities to the larger project. As a proof of concept, this distributed SDL undertook an effort to optimize the density and viscosity of electrolyte formulations. As part of the workflow, ontology and data interfaces were prepared at the Technical University of Denmark (DTU), SINTEF in Norway, and the École Polytechnique Fédérale de Lausanne in Switzerland; computer simulations of electrolyte formulations were performed at Dassault Systèmes in the United Kingdom and Germany, laboratory experiments were performed at Helmholtz Institute Ulm in Germany, and the ML optimizer was run at DTU. Although the experiment itself was rather simple compared with others detailed in this report, this research demonstrated the concept of ‘exposing laboratories as a service’, to improve utilization of facilities, equipment and capabilities, and maximize the return on investment of the funding spend on the development, construction and maintenance of these laboratories.

### 2.4. Biology

Recent advances and pioneering methods such as AlphaFold [44] demonstrate the potential of AI to advance the field of biology. Indeed, the abundance and complexity of large datasets within biology imply that high software-autonomy AIs are well suited for unravelling many of the mysteries of modern biology, either independently or as a complement to human researchers [45]. We describe notable SDLs for biological science research in this section and in table 5.

**Table 5.** Additional biological SDL publications of significance.

description	significance to SDLs	biology	hardware	software	reference
a robotic high- throughput screening system in a government laboratory was remotely connected to a corporate collaborator’s autonomous control system (2021)	optimization approach was successful and required testing only 7% of the variable combinations in the complete experimental space	an enzyme activity assay was optimized as a proof of concept for biological assay development	robot arm, liquid handler, fluorescence plate reader, automated plate washer	dynamic scheduling system. Inter-organization messaging protocol and system. Commercial LabView to generate experimental methods from requests. Bayesian optimization algorithm	[46]
an SDL to autonomously optimize retinal pigment epithelial (RPE) cell differentiation (2022)	impressive demonstration of extended culturing and manipulation of mammalian cells without contamination. The SDL tested 143 cell culture conditions in 111 days	RPE cell culturing parameters were optimized, resulting in 88% improved production	humanoid robot with robotic arms, micropipettes, CO_2_ incubator, microscope, aspirator, dry bath, sterile enclosure	AI image processing, batch Bayesian optimization algorithm	[47]
*BioAutomata* (2019), an SDL for microbial strain engineering	SDL performed autonomous assembly of DNA parts chosen by the design algorithm into plasmids, transformed the plasmids into bacterial cells, cultivated the bacteria, performed lycopene extraction and quantification	optimized lycopene production in *Escherichia coli* by autonomously designing experiments to vary the genetic elements driving pathway enzyme expression. Enhanced lycopene production 1.8-fold over 3 cycles from searching less than 1% of the variable space	iBioFAB system [48] with robot arm, liquid handler, thermocycler, colony picker, plate reader, centrifuge, incubator-shaker	‘acquisition policy’ algorithm decided on the genetic combinations to include in experiments based on results of previous cycles	[12]

The first example of a biology SDL was Adam [14], reported in 2009 by a team led by Ross King at Aberystwyth University and the University of Cambridge and described in §2.1. Adam was a closed-loop SDL with integrated hardware, software and ML algorithms. It could autonomously culture yeast, measure growth curves, vary growth medium ingredients and generate its own hypotheses about yeast functional genomics. Adam was challenged to identify certain unknown yeast genes encoding ‘orphan’ enzymes involved in amino acid biosynthesis. Adam was provided with a comprehensive logical model of the known metabolism of the base yeast strain, bespoke software to guide the SDL through the phases of the scientific method, and yeast strains deficient in various genes encoding known amino acid biosynthetic enzymes. Adam selected strains to grow and measure, conducted auxotrophic growth experiments, analysed results, and designed and performed new experiments based on those results. Adam successfully identified three genes encoding an orphan enzyme involved in lysine biosynthesis [14]. The seminal publication about Adam attracted substantial media attention, accompanied by exaggerated headlines. This prompted the late Bernard Dixon from the American Society for Microbiology to underscore in *Current Biology* that, while Adam did discover new scientific knowledge autonomously, the accuracy of the derived conclusions by Adam were predicated on being provided an accurate and extensive biological model [49].

In 2015, a multi-institute team led by Ross King debuted a new Level-4 robot scientist named Eve [15]. This SDL also devised and performed autonomous experiments with yeast expression of enzymes from other species as targets for chemical inhibition. Eve was challenged to discover lead compounds that selectively inhibit the dihydrofolate reductase gene from malaria parasites but not the human version of the enzyme. Instead of brute-force screening of libraries of thousands of candidate compounds, Eve first screened a small portion of the library and then used its ML software to derive quantitative structure-activity relationships (QSARs) from those results. Eve then autonomously decided which library compounds to screen in the next batch, based on the predictions from the QSAR model about their structures. Ultimately, Eve identified TNP-470 as a promising lead compound for malaria treatment.

A third SDL developed by King and colleagues, called Genesis, is currently under development [3]. As planned, this Level-4 system will be one of the most advanced SDLs for biology. Genesis will be used to autonomously conceive, plan, execute and analyse experiments to achieve a comprehensive understanding of yeast functional genomics and systems biology. Genesis is equipped with 1000 microbioreactors, an integrated mass spectrometry platform and an RNA sequencing system, allowing it to cultivate yeast and determine the metabolomic and transcriptomic states of each culture. The ML algorithms of Genesis will design and execute experiments with an impressive number of input parameters: approximately 20 000 yeast strains, thousands of culture conditions (combinations of growth-rate, optical density and growth medium additives) and input drugs (individuals or combinations from a collection of approximately 10 000 compounds). Genesis will autonomously measure and analyse growth rate, the levels of approximately 100 metabolites and the expression levels of approximately 6000 genes, for each culture. In 2019, a team led by Prof. King demonstrated a smaller scale proof-of-principle for this type of AI-powered systems biology model development using Eve to study yeast metabolic regulatory networks [50].

**Figure 1.**
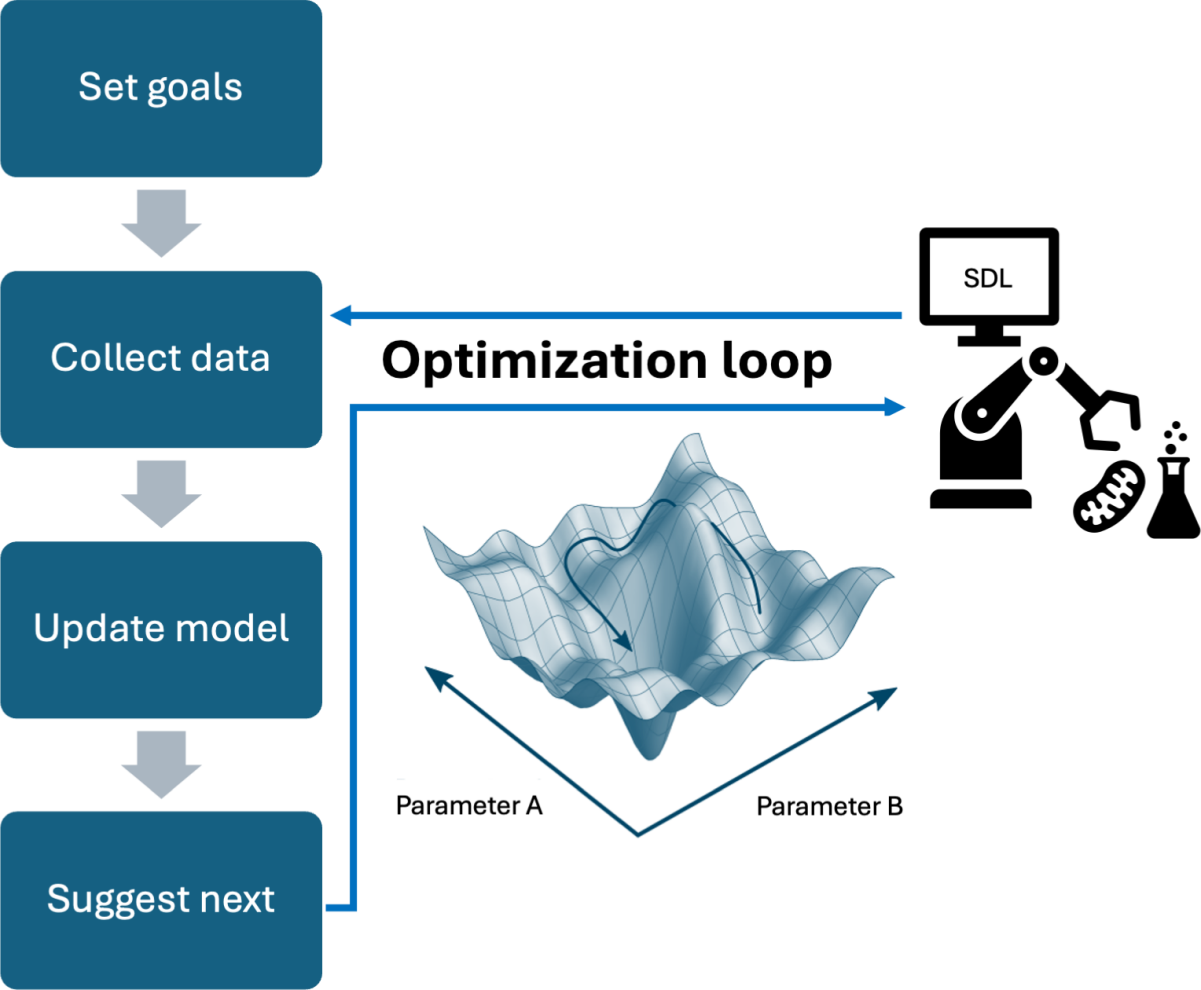
Optimizations represent the most common experiments performed using SDLs. Commonalities in the structure of these experiments and their tractability with general algorithms such as Bayesian optimization have contributed to their popularity in the SDL literature. Figure adapted from Hanaoka [51].

As we have seen, optimizations are the most common types of experiments performed with SDLs (see figure 1 and further discussion in §6). Optimization experiments tend to be less common in biology, which is perhaps one reason why there have been comparatively few reports of biology SDLs compared with chemistry or materials science, even though there exists a very well-developed commercial ecosystem of laboratory automation products and solutions for biological research. Many biology experiments also require protracted timeframes to generate results, especially for experiments involving genetic engineering or organism culturing. Extended, unattended experiments, especially those entailing repeated de-lidding of biological cultures, incur high risks of contamination or cross-contamination.

A classical type of biological optimization experiment is protein engineering, which requires balancing enhancement of certain protein properties such as enzymatic activity on a new substrate or stability to temperature or solvent with maintaining other properties such as expression level above a minimum threshold. An SDL for enzyme engineering was described in 2024 by a team led by Philip Romero, then at the University of Wisconsin [52]. In addition to serving as an excellent example of an SDL for enzyme engineering, the laboratory work for the following investigation was performed remotely by a subscription-access ‘cloud lab’. This aspect of the project is discussed in §2.5 below. Their SAMPLE (Self-driving Autonomous Machines for Protein Landscape Exploration) platform [52] leverages an intelligent agent that infers QSARs from experimental data, selects new protein sequences to test, directs the assembly of DNA fragments to generate the genes encoding the next round of enzymes, and analyses the results of enzyme thermostability assays for each round. The SAMPLE agent uses a Gaussian process model to predict whether protein sequences will be active or inactive. In their report, the scientists compared four different Bayesian optimization strategies for improving the thermostability of glycoside hydrolase family 1 enzymes. The team avoided complexities associated with culturing cells and then lysing them by using cell-free protein expression for the enzyme variants. Ultimately, SAMPLE identified enzyme variants that were at least 12°C more stable than the initial sequences by searching less than 2% of the full combinatorial landscape of mutations included in the experiment.

Researchers at Novartis upgraded an automated high-throughput system used to synthesize and characterize compounds for drug discovery into an SDL [16]. The SDL, which they named ‘MicroCycle’, can autonomously synthesize new compounds, purify them, perform chemical and biochemical assays with them, analyse the data and choose new compounds to synthesize and evaluate in the next cycle. MicroCycle is an impressively broad integrated drug discovery SDL, combining autonomous synthetic chemistry with *in situ* physicochemical, pharmacodynamic and biochemical assay capabilities. Reported in 2024, MicroCycle is perhaps the best-in-class platform for rapidly identifying and obtaining multidimensional data on pharmaceutical lead compounds.

FutureHouse is a philanthropy-funded venture established in late 2023 to develop ‘AI Scientists’ for biological research. They believe that AI Scientists can increase the experimental and analytical productivity of human scientists by 10- to 100-fold. FutureHouse is focusing on the AI ‘engine’ for biology, not on building an end-to-end automated laboratory. They view their in-house wet laboratory as a testbed where human scientists work on biological research and innovation projects together with AI Scientists to ‘discover concretely how AI will enable biology to scale’. [53] They elaborate, ‘Biology is the most unknown science, and is thus the perfect playground in which to determine, under conditions that are free from overfitting, whether an AI Scientist can make predictions, plan experiments, or conduct analyses at a superhuman level. At FutureHouse, integrated teams of machine learning researchers and biology researchers will iterate rapidly on constructing AI systems that can formulate hypotheses, plan experiments, reason mechanistically about the world, and apply those skills to concrete problems in biology’ [53].

### 2.5. Self-driving cloud labs

Several of the SDLs we have cited and described herein have remote-access features or networked architectures that connect geographically distributed teams and facilities. Some of these publications use the term ‘cloud’ or ‘cloud laboratory’ to denote these features or networks, which is understandable, if occasionally imprecise. In this report, we reserve the term ‘cloud lab’ for a remotely controlled lab-as-a-service that executes experiments according to the detailed commands of its subscribers, which they submit as lines of executable code [54–57]. Customers who fully utilize the capacity of their cloud lab subscriptions can process many more analytical samples per year than traditional labs (Emerald Cloud Lab provides a comparative example of 46 620 versus 8880, respectively [58]). Cloud labs thus offer a compelling value proposition for many researchers, as long as they can accept the drawbacks, such as difficulties inspecting precisely how samples were handled and troubleshooting failures. Cloud labs tend to be highly automated, but not exclusively so. Tasks too difficult or not worth the effort to automate are performed by lab technicians in as standardized and robot-like a fashion as possible. Although there appear to be few published accounts of AI-driven experiments performed in cloud labs, we consider the concept of self-driving cloud labs to be significant due to their low barriers to entry, democratization of access to laboratory capabilities and their accommodation of multiple ways for subscribers to incorporate AI or computational autonomy in the ‘intellectual’ aspects of their projects (see §2.1).

The SAMPLE platform [52] described in §2.4 is a notable example of research performed by a self-driving cloud lab. This SDL consisted of the autonomous ‘intelligent agent’ established by and located in the Romero laboratory and robotic workcells within the Strateos cloud laboratory. The agent performed design, modelling, data analysis, optimization and issuance of commands, and the cloud laboratory performed gene assembly, protein expression and biochemical assays of the proteins. Unfortunately, Strateos has since terminated public subscription-based access to their cloud lab and pivoted to a private on-premises cloud lab business model [59].

The second major published example of a self-driving cloud lab was a collaboration between the Gomes group at Carnegie Mellon University and Emerald Cloud Lab (ECL), one of, if not the, largest commercial cloud laboratories in the world. This publication describes Coscientist [60], an AI chemist that designs and plans complex experiments and generates ready-to-execute code in Symbolic Lab Language, the lingua franca of ECL and the cloud lab they built for Carnegie Mellon University [61]. Being partially based on the GPT-4 large language model from OpenAI, Coscientist features impressive chemical and general reasoning capabilities and an internet searching module that ‘significantly improves on synthesis planning’ [60].

It is possible that other subscribers are using autonomous AI systems to control their cloud lab experiments but have not published accounts of the work due to proprietary concerns. As thought-provoking as that possibility may be, an even greater step-change increase in the democratization of SDL technology would occur if commercial cloud laboratories began to offer their own autonomous AI agents in addition to subscription-based laboratory access, thus advancing past difficult-to-program lab-as-a-service to ‘tell the AI what you want in plain language’ SDL-as-a-service. Of course, democratization of any powerful technology can be disquieting. We address the safety and security concerns of self-driving cloud labs and SDLs in general in §4.

We believe that fully subscription-based self-driving labs are likely to emerge and that the primary question is ‘when,’ not ‘if’. For example, ECL instituted its own AI Scientific Advisory Board in 2023 [62]. If the AI agents of cloud laboratories are designed to assist users with moderate or even limited scientific skills and experience, are proficient at converting subscriber intentions expressed in plain language into executable code, and can perform data analysis autonomously, cloud lab subscriptions could potentially surpass current capacity. This would be transformational to the accessibility of research and development, and therefore, the entire enterprise of science; fundamentally, the only remaining barrier would be the subscription fees and materials costs.

### 2.6. Costs and challenges of SDL implementation

Establishing an SDL today requires substantial financial investment and technical expertise, particularly in hardware and software development. Specialized equipment for chemical handling, reaction execution, purification and analytical measurements can cost upwards of $1 million USD for off-the-shelf or customized commercial systems [63]. Vendor-supplied systems tend to include installation and setup, so they are usually operational shortly after delivery. Commercial scientific automation systems are ideal for predefined workflows, but may be insufficiently modular or reconfigurable for some laboratories. Mass-produced, general-purpose robots offer greater adaptability and price points as low as approximately $10 000 USD [64]. However, these systems require additional investment in software development and integration, and their experimental throughput is often lower than automated scientific systems. ‘Open hardware’ solutions, such as the FINDUS liquid handling workstation ($400 USD) [65] and Jubilee multi-tool gantry platform ($100–$2000) [66], represent ultra-affordable options for SDL hardware [67,68]. However, open hardware systems require assembly and integration by the user, which demands significant time and technical expertise. Additionally, a dearth of standardized protocols and robust user communities to support troubleshooting and development has further limited adoption of open hardware systems to a small set of laboratories willing to invest the time to reap their benefits [69,70].

The software required to coordinate automated workflows involving multiple instruments adds further complexity and cost. The primary reasons are that application programming interfaces tend not to be standardized across instrument manufacturers and sophisticated orchestration software is generally required to manage workflows in wet laboratories [63,71]. Combined with the limited programming expertise of typical physical and life sciences researchers, these barriers can restrict SDL accessibility, especially for smaller institutions and modestly funded labs. Cloud labs, discussed in the previous section, offer a potentially transformative option for establishing SDLs by eliminating the need to invest in hardware and the software to orchestrate and operate robotic or automated equipment. For example, establishing a physical or life sciences laboratory may require $800K USD for equipment with an annual maintenance cost of $80K USD. A cloud lab subscription consolidates these costs to monthly fees starting around $50K USD. Moreover, cloud lab subscribers can often jettison their own labs entirely and can focus their time and effort on science and, for SDL researchers, developing the autonomous AI ‘brains’ of their systems.

In terms of the AI and ML algorithms that underpin the software autonomy we consider so critical to the scientific potential of SDLs (discussed in §2.1), we observe a wide range of costs and implementation challenges across the SDL literature and community. At Levels 1 and 2 of software autonomy, the tasks performed by an SDL’s ‘AI brains’ are relatively straightforward and can be implemented using tools such as predictive models and dynamic workflow planners [72]. Dynamic workflow planners, such as AiiDA [73], automate task sequencing based on predefined rules or simple decision-making logic. These tools are often inexpensive or freely available, widely accessible, and can run on standard personal computers, with the primary challenges being the time and expertise required to install, configure and train the algorithms. By contrast, orchestration software, which is typically required for higher levels of autonomy, involves more complex coordination of multiple systems, processes and data streams. Orchestration software is often proprietary, expensive and resource-intensive, requiring specialized infrastructure and expertise to implement effectively. ChemOS [74] illustrates the extensive computational and software infrastructure required for advanced SDLs, which comes with high costs for equipment, licenses, in-house expertise and maintenance. At Level-3 SDLs, which are conditionally autonomous, more advanced optimization algorithms such as Bayesian optimization and active learning are needed to efficiently perform iterative cycles of the scientific method [75]. These methods require computational resources beyond standard personal computers, such as high-performance computing clusters or cloud-based platforms.

At software autonomy Level 4, SDLs must integrate cutting-edge techniques such as deep learning, generative models and natural language processing to autonomously generate hypotheses, execute protocols and analyse data [76–78]. Establishing the computational infrastructure for a Level-4 SDL from scratch is costly and time-intensive, requiring cloud computing subscriptions, software developers, computational experts and iterative development cycles spanning months or years. ChemCrow [79], for example, demonstrates how large language models can be augmented with chemistry-specific tools to autonomously observe, plan and execute actions. While ChemCrow leverages open-source tools, its implementation demands substantial computational resources and expertise.

The SDL space of today primarily consists of hardware and software developer-users because the market for commercial offerings, especially autonomous science AI software, is nascent. Eventually, we expect an even larger population of ‘pure’ SDL users or consumers (who lack the desire or ability to develop their own software or hardware) to emerge. As mentioned in the previous section, cloud labs including ECL may be working on commercial-grade autonomous science AI agents. Successful deployment of this technology would, we believe, catalyse the transformation of the SDL field from a developer-dominated ‘artisanal’ domain into a bona fide industry characterized by interoperable standards and mass production.

## 3. Intellectual property considerations of SDLs

### 3.1. Inventorship and conception

A key facilitator of technology commercialization in the modern world is the protection of intellectual property enshrined in national patent laws. A patent provides a legal basis for excluding others from practising an invention in a certain territory for a specified period. Title 35, Section 101 of the United States Code states, ‘Whoever invents or discovers any new and useful process, machine, manufacture, or composition of matter, or any new and useful improvement thereof, may obtain a patent therefor, subject to the conditions and requirements of this title’. U.S. Federal case law has held that ‘conception’ is the touchstone of inventorship for patent purposes, and that conception is, ‘the formation in the mind of the inventor, of a definite and permanent idea of the complete and operative invention as it is thereafter applied in practice’ [80,81]. Since conception occurs in the mind, it has been understood by the courts as only performed and performable by ‘natural persons’. It remains the consensus of the major patent offices of the world that AI systems are not eligible for inventorship or coinventorship credit or rights [82,83]. The U.S. and several other countries do allow for patenting AI-assisted inventions, as long as a natural person made a significant contribution to every claim [81,83].

**Figure 2.**
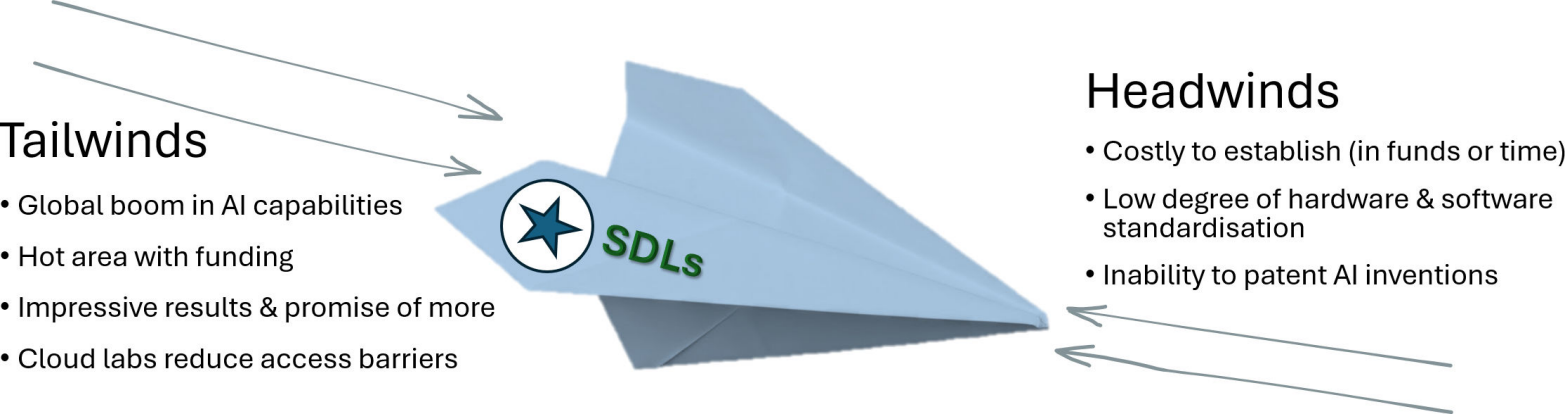
SDLs are currently subject to several countervailing forces, making it difficult to predict their trajectory, uptake and long-term impact. Although cloud labs presently require coding expertise, they increase access to SDLs by eliminating the need to establish or maintain one’s own laboratory facility.

This legal framework of conception and inventorship may represent a substantial ‘headwind’ (see figure 2) for SDLs with Level-3 or greater autonomy. Villasenor defines an ‘AI invention’ as, ‘an invention for which an AI system has contributed to the conception in a manner that, if the AI system were a person, would lead to that person being named as an inventor’ [5]. This is not merely a theoretical concept. AI systems and simpler algorithms have been generating novel inventions for years without conception by a human [5,84,85]. Early examples of such inventions from the mid-1990s include ‘*in-silico* evolved’ antennas with shapes created by genetic algorithms [86].

### 3.2. AI and SDL inventions under the law

Recently, an AI system named DABUS was reported by its creator, Stephen Thaler, to have invented a new type of flashlight and a novel container lid. Thaler sought to obtain patents for these inventions in several countries, naming DABUS as sole inventor, but the applications did not pass examination [5]. The U.S. Patent and Trademark Office (USPTO) ruled that the applications did not list a natural person as an inventor and were therefore incomplete. These rulings were upheld by two different U.S. courts, and the lack of a human inventor was also the rationale for rejection by the other countries [5]. An Australian court of appeals ruled in favour of Thaler in 2021, noting that an inventor is an ‘agent’ that could be a person or a thing, and that no provision of Australian patent law expressly refutes an AI system being an inventor, among other interpretations [87]. However, that decision was reversed the following year [88]. In 2022, the International Federation of Intellectual Property Attorneys submitted a response to a request for comment from the USPTO taking the position that, ‘AI is becoming powerful and creative enough to generate patentable contributions to inventions to which a human has arguably not made an inventive contribution but instead has directed the AI to endeavour towards the solution to a problem’ [81].

The issue of invention patentability is germane to a large cross-section of the AI space, not merely SDLs; however, SDLs embody perhaps the shortest and most direct connection between invention by AI and reduction to practice without human intervention. Furthermore, many of the legal analyses of the patentability of AI inventions reviewed for this study assume an engaged and involved human who continuously prompts and guides the AI system toward the ultimate invention(s) [5,82,85,89–91]. We have not seen legal consideration of a scenario in which human users input the objective function(s) and constraints of an experiment, then leave an SDL to perform multiple cycles of the scientific method, perhaps for weeks or months at a time, leading to inventions of which the users never conceived. In this sense, SDLs can be viewed as ‘invention machines’, and the patentability question as especially important to the SDL field.

The SDL literature is replete with examples of humans providing a few inputs to an SDL system: specification of the variable space of an experiment, some compositional and/or functional constraints, and an objective function to optimize, then leaving the SDL to autonomously design and perform multiple rounds of experiments using an adaptive search strategy. This process then culminates in the discovery of a novel chemical, material or protein variant that satisfies the original constraints, or a method or set of conditions for solving a problem. We have not yet seen how inventions generated this way can be patented [92]. An AI or other non-human entity cannot be named as an inventor, and natural persons assisted by AI systems may only be considered inventors if their contributions exceed what a person of ordinary skill could have made [80]. If standards for inventorship and patentability remain unchanged, continued advances in and expanded accessibility of AI could result in an unprecedentedly steep upward trend in the capabilities of the prototypical *person of ordinary skill in the art* [92]. This would further raise the bar for inventorship, such that ever fewer AI-assisted inventions would be patentable.

Excluding SDL-generated inventions from patent protection would likely reduce incentives for continued funding and investment in SDL development and adoption, ultimately limiting the economic and societal impacts of the field [5]. As Padmanabhan and Wadsworth note, ‘Why spend time and money on developing an AI that can generate a host of new technologies on its own if those technologies are not patentable by the individuals who made it possible?’ [85]

In lieu of being patented, inventions may be held as trade secrets; however, trade secrets offer much less protection from competition and have substantially less value to investors than patents [93]. Similarly, a case can be made that there is plenty of room for humans to file patents based on downstream research, optimization or applications of the molecules or materials invented by an AI or SDL; however, such patents would not protect against others profiting from the original AI- or SDL-generated inventions or applying them in different ways.

The issue of patentability is both intellectually captivating and profoundly important to the future of SDLs. It remains to be seen whether any nation will be willing to change its laws to provide a path to patent protection for inventions lacking the traditional elements of human conception.

## 4. Safety and security

SDLs are an emerging dual-use technology. As such, they may present both familiar and more unusual safety and security risks. Before SDLs achieve wider adoption and SDL-related goods and services become articles of commerce, an assessment of the potential risks posed by SDLs would inform the identification, development and deployment of any necessary safeguards. For the purposes of this discussion, the distinction between safety and security resolves to unintentional versus intentional harms, respectively, and the means to prevent, detect and mitigate them both. In the interest of maintaining the focus of this article on self-driving laboratories, we limit the discussion in this section to safety and security issues that, individually or in combination, are particular to SDLs, and avoid re-examining established concerns about laboratory automation of chemical or biological research [94], standard cloud labs [57] and the use of non-autonomous AI tools in research controlled by humans [95].

### 4.1. Risks

The fields of chemical and biological (CB) safety are concerned with accidental or unintentional events such as discharges of material or explosions in the laboratory that could result in harm to workers, external populations or the environment. Conversely, CB security focuses on prevention of deliberate releases or other intentional incidents such as bioterrorist attacks [96]. For chemistry and materials science, the primary risks are toxic emissions, fires or explosions. Fortunately, the typically small quantities of reagents handled by SDLs limits the scale of most incidents. For biological experiments, a primary risk is release of a pathogenic organism. Because organisms can self-replicate, working with small quantities in the lab may not limit the ultimate impact of a discharge.

Until recently, human professionals have been at the centre of the research enterprise. Every legitimate experimental research organization has at least one safety officer, and every researcher working in a lab undergoes safety training and has ultimate responsibility for their own safety and that of their colleagues. In the United States, mandatory safety regulations are promulgated by the Occupational Health and Safety Administration at the federal level. Biosecurity policy and practice are fostered in multiple domains, including law enforcement, the biosecurity enterprise of the federal government, research institutions and companies that market security products and services. Since deliberate incidents are, by definition, the result of conscious intent, CB security specialists are highly attuned to factors such as human psychology, access controls and the law and its enforcement.

At first blush, SDLs are disruptive to the CB safety and security status quo. In this research scenario, the machines are in control of experimentation, perhaps for weeks at a time. We consider the central questions at the core of SDL safety and security to be:

— How can an autonomous machine performing science experiments with hazardous materials or their precursors be sufficiently supervised and contained? (Safety)
— Is there potential that an SDL could ‘go off the rails’ and have its experimental objectives altered to more harmful or destructive ends? (Security)

Just as the levels of autonomy of SDLs were inspired by those conceived for autonomous vehicles (§2.1), the two questions above are close counterparts to the primary concerns about self-driving cars. Despite all the technology, design, redundancies and ‘training’ invested in vehicular autonomous systems, vehicles can encounter situations where they make a mistake that results in serious injury or fatality (Safety). A more odious fear is that of hackers infiltrating and sabotaging vehicle control software to cause collisions (Security).

The first question is essentially a minor extension of traditional laboratory safety. Due to human fallibility, inattention, fatigue, etc., SDLs have the potential to substantially enhance overall laboratory safety. Though not widespread at present, we encourage the developers of the next generation of SDLs to include the ‘standard safety feature’ of actively incorporating safety into their workflow by having their AI systems import and operationalize the relevant CB safety information for the experiments to be performed. This has not been a focus of the SDL literature; however, laboratory safety is not commonly discussed in scientific publications. On the other hand, while society may be prepared to cede substantial control of scientific experimentation to SDLs if the returns are beneficial, the public is likely not ready to leave CB safety to artificial intelligence alone.

The second question conjures scenarios of hacking as well as ‘sentient AI’ systems reminiscent of HAL 9000, the fictional computer from the 1968 film, *2001: A Space Odyssey*, that decided to kill the crew of astronauts. Technical articles about remotely controlled laboratories do tend to include a section about cybersecurity features and their importance. In modern AI parlance, this scenario would be described as an AI achieving ‘autonomous replication in the real world’ [97]. While the majority of the AI systems controlling SDLs described in the current literature are highly specialized for planning and executing their experiments, and do not seem remotely capable of ‘escaping’ from their source code or designed constraints, it is entirely possible that more complex, less-understood, general-purpose models could become the norm for running SDLs in the near future. This would come with an increased risk of autonomous deviation from preset objectives.

Self-driving cloud labs carry some additional safety and security concerns due to the separation of the laboratory, both in distance and organizationally, from the controlling AI system or the human team in charge of the experiment. For example, consider errors in the AI-generated cloud lab execution code causing materials to be mislabelled or misloaded, resulting in noxious reaction products. If the errors result from unintentional causes, this is a safety issue. If they stem from saboteurs or the AI gone rogue, it is a security incident. In either case, a primary difference is that it is harder to observe or track the contemporaneous happenings of a remote cloud laboratory than the activities within a traditional laboratory. This makes cloud laboratory experiments less accessible to direct observation by a knowledgeable person, such that timely intervention to prevent a mishap is less likely. The remote location of cloud laboratories may, in some cases, enhance their attractiveness as a target for sabotage or worse [57]. This is not unique to SDLs; however, imagine a cloud laboratory that offered an SDL service based on its own, centralized AI system. If this controlling AI became set itself towards a malevolent objective and could defeat the cloud laboratory’s cybersecurity controls, the rogue AI could rewrite the code for multiple customer experiments to create harmful products or dangerous conditions. Such an ‘SDL as a service’ has yet to be made available to the public; however, Emerald Cloud Lab has declared its intention to implement AI within its environment [98] and collaborated on the recent publication describing Coscientist (described in §2.5), an AI system that ‘autonomously designs, plans, and performs complex [chemistry] experiments’ [60]. This publication included a ‘dual-use study’ within its supplementary information package that summarizes attempts to task the AI system with devising synthetic routes to illicit compounds. Within this study, the authors remarked, ‘the system significantly reduces the entry barrier for ill-intentioned low-knowledge actors as they could conduct malicious experiments without any prior training. While the Intelligent Coscientist’s capabilities of running scientific experiments raises [sic] real concerns for the potential of dual use, fully monitored cloud labs remain a safer choice than simply remote-connected machines. Screening, monitoring, and control safety systems such as the ones implemented by major cloud labs offer an additional layer of protection from potential misuses or bad actors’.

Commercial cloud labs are, given their desire for self-preservation at minimum, likely to offer more protection against inappropriate use than unattended or remotely accessible SDLs. Overall, the major entities in the SDL space appear to be seriously and genuinely concerned with safety and security.

### 4.2. Recommendations for prevention and mitigation

Human oversight of SDLs is a key element of their safety and security policies and procedures, given the present states of society and SDL technology. We are in an environment of rapidly accelerating AI capabilities, several of which are already struggling to be accepted by society as aligned with the interests of humanity. It is prudent and even benefits the self-driving laboratory field to ensure that whenever an autonomous experiment is run, humans with knowledge of the experiment are held responsible and accountable for its safe and secure execution. This means that the responsible humans review and approve all experimental plans and executable code. We consider this human review and approval so critical to the long-term acceptance of SDLs that we implore SDL developers to institute software features such as visual symbols and concise plain language summaries to make this process as easy as possible. This review should consider the safety characteristics of chemicals and other raw materials; detection, containment and safe shutdown measures for spills and related mishaps; the sequences of biological molecules and identities of biological strains; and the handling, mixing and disposal of materials throughout the project. Importantly, SDL systems should be strictly compartmentalized to prevent alteration of those plans and code after human approval.

It is appropriate that leading organizations in the laboratory safety space such as the Laboratory Safety Institute and The Association for Biosafety and Biosecurity develop and publish guides for safely working with SDLs, including examples of safety documentation, hazards analyses and training materials.

In terms of security, in addition to following the highest standards of AI containment [97], cybersecurity, chemical security and biosecurity, organizations operating SDLs should first ensure that monitoring and alerting systems capable of detecting unauthorized access, unanticipated production or release of hazardous materials, and other highly consequential incidents are available for deployment in SDL settings. Second, SDL owners and operators should ensure that a human supervisor is available and empowered to pause or terminate any autonomous experiment if they detect evidence of malicious behaviour or the proclivity therefor (e.g. with a ‘kill switch’). Such evidence would include attempts by the AI system to conceal, falsify or obfuscate experimental details. All events of this type should also be reported to relevant authorities for potential investigation. Finally, if proven to provide a clear benefit to the safety and security of these systems, technical safeguards for SDLs should be standardized and their use potentially mandated. The best way to handle safety and security incidents in laboratories is to prevent them from occurring.

Failure to institute sensible, widespread policies and procedures intended to prevent adverse events or to catch them early risks obstruction of the entire SDL field in reaction to even one high-profile safety failure or security breach. Following a policy of ultimate human awareness and accountability for the actions of SDLs is a key safeguard for ensuring that this technology will continue to develop and thrive. AI technology is simply not yet sufficiently trustworthy to leave safety and security under its charge. Just as importantly, ultimate human responsibility ensures that liability for harms caused by SDLs remains with their human users or creators. Liability for damages under the law is a powerful deterrent, and thus, key form of governance for SDL construction and use. No legal system holds machines liable for damages [99], so it is vital to uphold a close connection between humans and the actions of SDLs to prevent incidents from being attributed to mere ‘AI or machine failure’, which would encumber the pursuit of legal recourse for those harmed.

## 5. Potential impact: labour force

A key characteristic of technological revolutions has been their massive reorganization of labour markets and forces. As has occurred with other disruptive technologies [100], many pundits and lay people anxiously predict that AI and automation will displace many types of jobs across the economy [101,102], rendering millions of workers unemployed or even unemployable without costly retraining. On the other hand, innovation has historically led to economic growth [100], and new technologies also create entire new, previously unimagined types of jobs. These include social media marketing coordinators, independent influencers and video bloggers making a living by leveraging the direct-to-consumer connections made possible by the internet and its applications. Indeed, fully 60% of U.S. employment in 2018 was in job specialties that did not exist in 1940 [103]. Acemoglu and Restrepo argued in 2018 that, unfortunately, economists were ‘far from a satisfactory understanding of how automation in general, and AI and robotics in particular, impact the labour market and productivity’ [102]. According to their framework, technologies like automation and AI, i.e. the foundations of SDLs, will certainly displace labour for tasks that are readily automatable; however, the increased productivity associated with this displacement tends to increase labour demand for other, less automatable jobs, due both to increased spending power and the automation technologies themselves, which must be developed, maintained and serviced [104].

Although many economists study the interplay between technology and labour from a macro perspective, there are few recent reports that focus on the dynamics of the scientific and engineering workforce in response to new, potentially job-displacing technologies. Nevertheless, in addition to the general labour market principles taught by Acemoglu and Restrepo, we discovered several other contemporary studies [100,101,105–107] that searched for and found no evidence of broad-based displacement by AI of high-skill jobs with high education requirements, such as research scientists and engineers.

### 5.1. Some labour statistics

According to the U.S. Bureau of Labor Statistics (BLS), in 2023 there were 16 500 chemists (BLS occupation code 19-2031), 2860 materials scientists (code 19-2032), 6780 microbiologists (code 19-1022) and 21 120 biochemists and biophysicists (code 19-1021) employed in Scientific Research and Development Services [108]. These are the four primary fields of research employing SDLs, with the first two representing the lion’s share of examples in the literature. Rounding up to the nearest thousand, the total number of research and development scientists in these four categories was about 48 000.

For perspective, customer service has been identified as an occupation especially prone to disruption by AI [107,109]. There were 2 858 710 customer service representatives (code 43-4051) working in the United States in 2023 [108]. By virtue of these population figures alone, we see that the impact of job displacement by SDLs on the overall U.S. economy would be small compared with the decimation of much more populous occupations likely to be impacted by AI. Additionally, scientific research has not been identified as a profession with a high propensity for replacement by AI, so the fraction of research jobs likely to be displaced is also bound to be lower than that for customer service workers and other identified roles (e.g. translators, radiologists [107,109]).

### 5.2. Labour effects are difficult to predict

Ross King, a founding father of the SDL field, told the authors during an interview that some of his motivation for developing a ‘robot scientist’ came from seeing empty, inactive laboratories as he departed from work every evening. He wondered if robots and computers could increase the productivity and the return on investment of science by utilizing all hours of the day. Prof. King never imagined that SDLs would put scientists out of work and does not think that is likely to happen in the short term. Even for very widespread and disruptive technologies, whether they will supplant certain professions or increase the productivity and inherent value thereof has been a challenge to forecast. For example, automated teller machines were predicted to supplant the majority of bank tellers, but instead, the United States now has many more bank tellers (at many more bank branches) performing a largely different set of tasks, because the machines are poorly suited to developing relationships with customers [110]. Autor *et al.* determined that new technologies can impact worker tasks by automating them or augmenting them. Occupations for which a high proportion of tasks become automated, such as radiologic technologists or machinists, experience a reduction in labour demand and employment. Conversely, professions with more augmented than automated tasks, such as industrial engineers and analysts, can experience employment growth [101]. The central question for scientists, then, is whether SDLs will be more of an automating or augmenting force. In an interview, Prof. King surmised to us that in the nearer term (approx. 10 years), as SDLs continue to be developed and demonstrate their value, they will most likely serve as productivity ‘force multipliers’ for scientists (augmenting force) than as labour displacers (automating force). The increase in data production and experimental throughput associated with SDLs running nearly round the clock will alter the mix of tasks for junior researchers, who typically perform most of the repetitive work in laboratories. This increased productivity will raise standards and expectations of output per researcher. Though increased wages may not necessarily follow, other benefits such as elevated job satisfaction and greater access for individuals with physical disabilities to the profession may be realized. For the foreseeable future, human scientists will still be required to develop research questions and initial hypotheses, write and publish papers, serve as peer reviewers, compose applications for funding and network with those who control the resources.

In our world of limited public resources for scientific research, the question of how such funds are earmarked deserves some attention. The ascent of SDLs naturally brings the concern that a substantial portion of the funds that would have previously been allocated for students and trainees might be diverted to construction, operation or subscriptions to SDLs—especially if certain productivity metrics appear to support these decisions. Fortunately, this concern is largely being raised in advance. We believe it would be short-sighted and ultimately detrimental to the progress of science and technology to disadvantage the next generation of human talent this way. We strongly recommend that the community, especially funding bodies, maintain a robust emphasis on supporting education and training. After all, keeping up with rapid advances in the capabilities and productivity of SDLs will almost surely require an even more scientifically literate and adroit community of research professionals than we have today.

Should SDLs become widespread enough to replace, say, a quarter of the research and development scientists in the categories listed above, that would most likely imply the SDLs were phenomenally successful at creating scientific, engineering and economic value through efficiency, ingenuity or a combination thereof. This growth would potentially create new opportunities for such displaced scientists (and perhaps many more professionals) to assume equally rewarding roles converting and scaling nascent SDL discoveries into groundbreaking new products and services.

## 6. Conclusions

Despite our substantial efforts to research the past and present of self-driving laboratories, their future trajectory, popularity, capabilities and scientific impact appear uncertain. We believe our dearth of predictive confidence stems from the current exposure of the technology to numerous counteracting forces. Figure 2 depicts an aeronautical analogy of the state of SDLs. While they are experiencing multiple ‘tailwinds’ propelling them into the future of research and development, they also face ‘headwinds’ that could slow their progress or even stall it altogether. These forces can be expressed as questions, such as, Are we ready to fundamentally change the ways we work with computers, software and robots to do science? Are our legal and intellectual property systems ready for AI-generated inventions with no human coinventors? Are we ready to stay ahead of developments in autonomous AI and self-driving laboratories to mitigate their evolving risks to our safety and welfare?

In her 2021 paper, *Why AI is Harder Than We Think* [21], Melanie Mitchell revealed a form of Moravec’s paradox [111] about artificial intelligence: many things that humans find easy and routinely perform without conscious thought, such as walking in a crowd, identifying and naming the objects in our visual field, or having a conversation, are among the hardest challenges for machines, whereas many of the toughest tasks for humans, such as playing chess or translating between hundreds of languages, are rather easy for machines. A parallel with SDLs can be observed, relating to our discussion of software and hardware autonomy in §2.1. Advances in robotics for laboratory automation may be challenging to achieve and impressive when implemented, but the majority of automated laboratory tasks fall in the ‘easy for humans’ category (for highly repetitive tasks, the challenge for humans is usually the repetition, not the task itself). On the other hand, while most humans require years of formal education to learn to think like scientists and assimilate a modest quantity of scientific knowledge, AI systems can rapidly consume and process entire corpuses of technical literature, draw profound connections and inferences, and pose original scientific questions and hypotheses.

As mentioned in §2.4 and figure 1, it is apparent that most present-day SDLs have been designed and employed to perform optimization experiments within a defined variable space. This is understandable, since optimizations are highly structured, well-defined experiments for which an extensive library of algorithms has been developed [112]. To be sure, optimizations are an important class of scientific and engineering endeavour. However, this library of algorithms means that optimizations have largely been reduced to rote iteration of mapping and searching cycles. For these reasons, we consider that optimization experiments can justifiably be viewed as somewhat ‘low-hanging fruit’ for SDLs. What excites us more is the prospect of SDLs being used to design and carry out more paradigm-shifting experiments that capitalize on the intellectual strengths of AI systems mentioned above. Efforts such as Genesis [3] and at FutureHouse [53] appear to offer glimpses of the more sophisticated capacities for reasoning, learning, and inquiry that subsequent generations of SDLs will possess.

The intellectual complementarity of AI and human scientists is what provides us the greatest inspiration and optimism for the development of subsequent self-driving laboratories with the potential to profoundly and qualitatively transform science. AI systems process information and solve problems differently than humans [2], and their ability to complement or vastly exceed human aptitude in certain areas of science, such as the design of novel proteins and the prediction of their three-dimensional structures, has already been demonstrated [113]. If SDL technology is judiciously shepherded, we can envision a near future in which autonomous systems and human scientists work together in a synergistic, symbiotic fashion that capitalizes on the unique strengths of the other to advance knowledge and address crucial problems facing our planet.

## Data Availability

This article has no additional data.
